# Synthesis and evaluation of fluorine-18 labelled tetrazines as pre-targeting imaging agents for PET

**DOI:** 10.1186/s41181-024-00250-6

**Published:** 2024-03-06

**Authors:** Eva Schlein, Johanna Rokka, Luke R. Odell, Sara Lopes van den Broek, Matthias M. Herth, Umberto M. Battisti, Stina Syvänen, Dag Sehlin, Jonas Eriksson

**Affiliations:** 1 Department of Public Health and Caring Sciences, Uppsala University, 751 85 Uppsala, Sweden; 2 Department of Medicinal Chemistry, Uppsala University, 751 23 Uppsala, Sweden; 3 Department of Drug Design and Pharmacology, University of Copenhagen, 2100 Copenhagen, Denmark; 4 Department of Clinical Physiology, Nuclear Medicine & PET, Rigshospitalet Copenhagen University Hospital, Blegdamsvej 9, 2100 Copenhagen, Denmark; 5 PET Centre, Uppsala University Hospital, 751 85 Uppsala, Sweden

**Keywords:** Inverse electron demand Diels–Alder reaction, IEDDA, Pre-targeting, Tetrazine, Trans-cyclooctene, TCO, Alzheimer’s disease, Bioorthogonal, Fluorine-18, PET

## Abstract

**Background:**

The brain is a challenging target for antibody-based positron emission tomography (immunoPET) imaging due to the restricted access of antibody-based ligands through the blood–brain barrier (BBB). To overcome this physiological obstacle, we have previously developed bispecific antibody ligands that pass through the BBB via receptor-mediated transcytosis. While these radiolabelled ligands have high affinity and specificity, their long residence time in the blood and brain, typical for large molecules, poses another challenge for PET imaging. A viable solution could be a two-step pre-targeting approach which involves the administration of a tagged antibody that accumulates at the target site in the brain and then clears from the blood, followed by administration of a small radiolabelled molecule with fast kinetics. This radiolabelled molecule can couple to the tagged antibody and thereby make the antibody localisation visible by PET imaging. The in vivo linkage can be achieved by using the inverse electron demand Diels–Alder reaction (IEDDA), with trans-cyclooctene (TCO) and tetrazine groups participating as reactants. In this study, two novel ^18^F-labelled tetrazines were synthesized and evaluated for their potential use as pre-targeting imaging agents, i.e., for their ability to rapidly enter the brain and, if unbound, to be efficiently cleared with minimal background retention.

**Results:**

The two compounds, a methyl tetrazine [^18^F]MeTz and an H-tetrazine [^18^F]HTz were radiolabelled using a two-step procedure via [^18^F]F-Py-TFP synthesized on solid support followed by amidation with amine-bearing tetrazines, resulting in radiochemical yields of 24% and 22%, respectively, and a radiochemical purity of > 96%. In vivo PET imaging was performed to assess their suitability for in vivo pre-targeting. Time-activity curves from PET-scans showed [^18^F]MeTz to be the more pharmacokinetically suitable agent, given its fast and homogenous distribution in the brain and rapid clearance. However, in terms of rection kinetics, H-tetrazines are advantageous, exhibiting faster reaction rates in IEDDA reactions with dienophiles like trans-cyclooctenes, making [^18^F]HTz potentially more beneficial for pre-targeting applications.

**Conclusion:**

This study demonstrates a significant potential of [^18^F]MeTz and [^18^F]HTz as agents for pre-targeted PET brain imaging due to their efficient brain uptake, swift clearance and appropriate chemical stability.

**Supplementary Information:**

The online version contains supplementary material available at 10.1186/s41181-024-00250-6.

## Background

Brain imaging with positron emission tomography (PET) is an important diagnostic tool for studying neurodegenerative conditions such as Alzheimer’s disease and Parkinson’s disease. A PET investigation is performed by intravenously administering a PET radioligand, a compound labelled with a positron-emitting isotope, after which its distribution in space and time in the body is mapped using a PET-scanner. Brain imaging is typically performed with PET tracers having a low molecular weight and relatively high lipophilicity which facilitate efficient passage over the endothelial cell layer of the blood–brain barrier (BBB). However, to achieve optimal imaging results, it is important to obtain a high target-to-background ratio inside the brain by maximizing the specific binding to the target while minimizing the background signal through fast clearance of non-binding fractions from the brain and the blood.

Antibodies are highly specific to their target and are therefore attractive tools to be used in functional imaging. Their specificity to their epitope allows for the distinction of various aggregation entities of amyloid-beta (Aβ), (Magnusson et al. 2013; Sehlin et al. 2017; Syvänen et al. 2022; Sehlin et al. 2019) which can open up new possibilities for immune imaging, including the specific targeting of soluble or diffuse Aβ aggregates. The recent approval of the therapeutic Aβ antibody Lecanemab (Dyck et al. 2023) has increased the need for relevant immune tracers for target screening and treatment monitoring. While larger molecules, e.g. antibodies and antibody fragments, used in PET imaging can be more selective and resistant to metabolic degradation than small molecules, they tend to have slower kinetics and limited brain distribution (Swiercz et al. 2014). This is due to the restrictions of the BBB, which affects the distribution from the systemic circulation into the brain parenchyma (Chowdhury et al. 2021). Therefore, while clinically useful for cancer imaging and other peripheral targets, (Liberini et al. 2021; Dongen et al. 2021; Chomet et al. 2021; Wei et al. 2020) large molecules have been less explored in applications involving imaging of brain targets.

One approach to overcome these limitations has been to fuse antibodies with a molecule that interacts with the transferrin receptor (TfR). Such bispecific formats have previously been described to display brain concentrations up to 100-fold higher than those seen with unmodified IgG antibodies (Hultqvist et al. 2017). Another approach is to use a two-step pre-targeting technique, where an antibody modified with a coupling tag is administered first and allowed to accumulate at the target site and clear from the circulation. This is followed by administration of a small rapidly clearing radiolabelled molecule that couples to the tagged antibody at the target site in vivo (Stéen et al. 2018). Given the low concentrations of both the antibody and the radiolabelled molecule in vivo, a fast and efficient bond formation is necessary. Pre-targeting systems, such as streptavidin–biotin or antibody-hapten, have been explored thoroughly and can sometimes cause immunogenicity (Rossin and Robillard 2014), which is why bio-orthogonal functionalisation may be a preferred alternative (Sletten and Bertozzi 2009). Bio-orthogonal reactions are described as chemical reactions which can occur inside living organisms without interference from other functional groups within the organism (Knight and Cornelissen 2014; Prescher and Bertozzi 2005).

The inverse electron demand Diels − Alder (IEDDA) reaction irreversibly forms covalent bonds between an electron‐rich dienophile and electron‐poor diene, e.g. coupling of *trans*-cyclooctenes (TCOs) with tetrazines (Pagel 2019). Specifically the tetrazine ligation reaction has shown potential for clinical use due to its bio-orthogonal nature, the irreversible bond formation between TCOs and tetrazines, and specifically because of the fast reaction rate of up to 10^7^ M^−1^ s^−1^ (Ravasco and Coelho 2020; Rondon and Degoul 2020). Combining the fast and modular properties of the tetrazine ligation reaction with the specificity of antibodies is a potent approach, particularly when using a short-lived radionuclide like fluorine-18, which has a high percentage of positron decay, suitable beta energy for high resolution imaging and an excellent half-life for clinical applications (Jacobson et al. 2015).

Pre-targeting strategies with the tetrazine ligation reaction is a promising approach for brain imaging, (Bredack et al. 2022; Shalgunov et al. 2022; Lopes van den Broek et al. 2022; García-Vázquez et al. 2022; García-Vázquez et al. 2021) and tetrazines for peripheral pre-targeting have often been characterised by low lipophilicity to reduce nonspecific binding and to promote fast clearance (Keinänen et al. 2016). However, decreasing the lipophilicity of small molecules can also decrease BBB permeability, so it is important to consider this trade-off when selecting compound candidates for pretargeting within the brain.

The aim of this study was to assess the brain kinetics of two new ^18^F-labelled tetrazines [^18^F]MeTz (**6a**) and [^18^F]HTz (**6b**), developed for pre-targeting and brain imaging. These compounds were designed to balance synthetic feasibility with regards to both precursor and ^18^F-incorporation with physicochemical properties (cLogP < 4, molecular weight < 450 g/mol, number of hydrogen bond donors < 3) consistent with expected BBB penetration (Xiong et al. 2021). Notably, the precursors were synthesized from readily available starting materials, thereby expanding the repertoire of previously reported ^18^F-labeled agents (García-Vázquez et al. 2021; Battisti et al. 2021).

## Methods

### *Synthesis of [*^*18*^*F]MeTz 6a and [*^*18*^*F]HTz 6b*

**Scheme 1:**
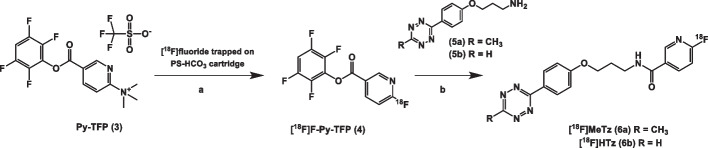
[^18^F]Fluoride was trapped on a Chromabond PS-HCO_3_ cartridge and dried with acetonitrile. **a** The precursor Py-TFP 3 in acetonitrile was reacted with the [^18^F]fluoride on the solid support and the formed [^18^F]F-Py-TFP 4 ester eluted from the cartridge. Unreacted precursor Py-TFP 3 was removed by an Oasis MCX Plus cartridge. **b** The labelled tetrazines [^18^F]MeTz 6a and [^18^F]HTz 6b were synthesized by direct amidation

The synthesis of Py-TFP **3**, Scheme 1, was performed as previously described (Olberg et al. 2010) with minor modifications (Additional file 1). Compounds [^18^F]MeTz **6a** and [^18^F]HTz **6b** were radiolabelled in a two-step procedure. Cyclotron-produced [^18^F]fluoride in an aqueous solution (16–20 GBq) was concentrated and trapped on a QMA cartridge (Chromabond PS-HCO_3_ Shorty/45 mg, Macherey–Nagel). The [^18^F]fluoride was then reacted on the solid support with a solution of Py-TFP **3** (10 mg, 30 μmol) in acetonitrile (0.8 mL), which was passed over the QMA cartridge followed by neat acetonitrile (0.7 mL). Both solutions were passed over the cartridge at a flow rate of 0.4 mL/min. The reaction formed **4** at room temperature, which was then released with the flow into a teflon tubing acting as a reservoir. After the elution was completed, the collected solution in the teflon tubing was pushed with air over an Oasis MCX Plus Short cartridge (Waters), preconditioned with acetonitrile (3 mL), to remove unreacted cationic precursor **3**.. The purified solution containing [^18^F]F-Py-TFP was then delivered to a septum-equipped vial (5 mL) containing 3-(4-(6-methyl-1,2,4,5-tetrazin-3-yl)phenoxy)propan-1-amine hydrochloride **5a** or 3-(4-(1,2,4,5-tetrazin-3-yl)phenoxy)propan-1-amine hydrochloride **5b** (1–2 mg, Broadpharm, San Diego, USA) and DMSO (0.1 mL).

Triethylamine (5 µL) in acetonitrile (100 µL) was added to the mixture, and the mixture was heated for 15 min at 55 °C. Following dilution of the mixture with a 0.1% v/v aqueous solution of trifluoroacetic acid (3 mL), the labelled tetrazines were purified by semi-preparative HPLC on an ACE C18 5 µm (150 × 10 mm) column. The HPLC mobile phase consisted of a 59:41:0.01% v/v ratio of water, ethanol and TFA, with a flow rate of 5 mL/min. The column eluate was monitored using a UV detector (254 nm) and a radiodetector. The retention times for [^18^F]MeTz **6a** and [^18^F]HTz **6b** were 12 min and 8 min, respectively.

The collected HPLC fraction containing the labelled tetrazine was reformulated in ethanol (0.5 mL) using the conventional procedure, which included dilution with water (20 mL), fixation on a Sep-Pak tC18 Plus Light cartridge, a rinse with water (20 mL) and air prior elution with the ethanol (0.5 mL) into a septum equipped vial (1 mL).

The radiochemical purity of the labelled tetrazines were assessed by analytical HPLC using a Chromolith Performance RP-18e column (4.6 × 100 mm, Merck). The eluent for [^18^F]MeTz **6a** was a mixture of water and acetonitrile in a 75:25 ratio, and for [^18^F]HTz **6b** a mixture of water and acetonitrile in a 65:35 ratio. The flow rate was 4 mL/min. The retention times of [^18^F]MeTz **6a** and [^18^F]HTz **6b** were 4.5 min and 8 min, respectively, and the radiochemical purities were 97% and 96.5%. The identity of the labelled tetrazines was confirmed by co-injecting and matching retention times with isotopically unmodified reference compounds.

### Synthesis of reference compounds *10a* and *10b*

6-Fluoronicotinic acid **7** (1.0 g, 7.2 mmol), fluorophenol (1.1 g, 9.5 mmol) and N’,N’-dicyclohexylcarbodiimide (1.36 g, 6.5 mmol) were dissolved in acetonitrile (100 mL) and the mixture was stirred at room temperature overnight. The mixture was then filtered, and the filtrate was allowed to stand at 4 °C overnight. After a second filtration, volatiles were removed under reduced pressure. The residue was dissolved in a small amount of hot hexane and immediately filtered. The filtrate was stored at 4 °C overnight, filtered and washed with cold hexane. The resulting 0.82 g (2.8 mmol) 6-fluoronicotinic acid 2,3,5,6-tetrafluorophenyl ester **8** was collected as white powder.

3-(4-(6-methyl-1,2,4,5-tetrazin-3-yl)phenoxy)propan-1-amine HCl salt **9a** (1.5 mg, Broadpharm, San Diego, USA) and 3-(4-(1,2,4,5-tetrazin-3-yl)phenoxy)propan-1-amine HCl salt **9b** (1.5 mg), respectively, were predissolved in DMSO (0.1 mL). The tetrafluorophenyl ester **8** (2.0 mg) in acetonitrile (1 mL) and triethylamine (5 μL) was added and resulting mixture was heated at 55 °C for 3 h.

**Scheme 2:**
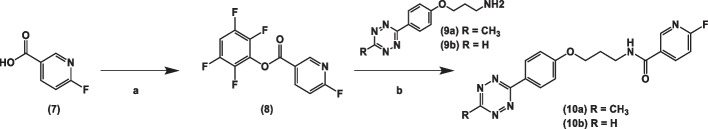
**a** 6-fluoronicotinic acid was reacted with TFP and N,N-dicyclohexylcarbodiimide in acetonitrile over night at RT to form 6-fluoronicotinic acid 2,3,5,6-tetrafluorophenyl ester **b** The reference compounds were synthetized by direct amidation

The tetrazines **10a** (6-fluoro-N-(3-(4-(6-methyl-1,2,4,5-tetrazin-3-yl)phenoxy)propyl)nicotinamide)and **10b** (N-(3-(4-(1,2,4,5-tetrazin-3-yl)phenoxy)propyl)-6-fluoronicotinamide), Scheme 2, were purified by semi-preparative HPLC on a Kinetex C18 5 μm (150 × 10 mm) column. The HPLC mobile phase consisted of a 59:41:0.01% v/v ratio of water, ethanol and TFA, with a flow rate of 5 mL/min. The column eluate was monitored using a UV detector (254 nm). The retention times for the corresponding compounds were 12 min and 8 min, respectively.

The collected HPLC-fractions containing **10a** and **10b** in HPLC eluent were extracted with dichloromethane (50 mL). The organic phase was collected and dried with anhydrous magnesium sulphate, filtered and the solvent was removed under reduced pressure. The resulting products were isolated as pink solids. The structures were confirmed via NMR analysis (Additional file 1).

### Animals

Transgenic mice (tg-ArcSwe), 14–20 months old (n = 4), maintained on a C57BL/6J background, and age-matched wild-type (WT) mice (n = 4) were used in this study. Tg-ArcSwe mice express the human amyloid-β precursor protein (*AβPP*) with the Arctic (*AβPP E693G*) and Swedish (*AβPP KM670/671NL*) mutations under the murine Thy1 promoter, resulting in Aβ pathology developing from the age of 6 months (Lord et al. 2006). All procedures were approved by the Uppsala County Animal Ethics board (5.8.18–20,401/20), following the rules and regulations of the Swedish Animal Welfare Agency, and were in compliance with the European Communities Council Directive of 22 September 2010 (2010/63/EU).

### PET experiments

Mixed pairs of mice (tg-ArcSwe and WT) were anesthetised with sevoflurane 4.0% and placed in a prone position in a Mediso NanoPET/MR (Mediso Medical Imaging System, Hungary). The anaesthesia was maintained during the study using 3.5–4.0% sevoflurane in a 0.5 L/min flow of 50% oxygen and 50% medical air. Compound [^18^F]MeTz 11.3 ± 1.2 MBq or compound [^18^F]HTz 10.4 ± 1.5 MBq were injected i.v. in the tail vein. The PET acquisition time in the PET scanner was 60 min with a field of view (FOV) of 9.8 cm. Following PET, a 5 min CT scan was acquired using a Mediso NanoSPECT/CT (Mediso Medical Imaging System). After scanning, the mice were perfused with 0.9% NaCl for 3 min intracardially. The organs were isolated and the radioactivity in the organs was measured with a wizard gamma counter (PerkinElmer, Turku, Finland). The radioactivity concentrations, quantified as standardized uptake value (SUV), were calculated as follows:

$$SUV=\frac{\mathrm{Measured\, radioactivity \,per \,gram\, tissue}}{\mathrm{Injected\, radioactivity\, per\, gram\, bodyweight}}$$

The PET data was reconstructed on a 160 × 160 × 128 grid with 0.5 × 0.5 × 0.6 mm^3^ voxels using 3-dimensional ordered-subsets expectation maximisation (20 iterations) into the following frames: 10 × 2 s, 2 × 5 s, 3 × 10 s, 2 × 30 s, 3 × 60 s, 5 × 300 s and 3 × 600 s. The CT raw files were reconstructed using filtered back-projection. Processing of the PET and CT images was performed with Amide, version 1.0.4 (Loening and Gambhir 2003). The CT scans were manually aligned with a T2-weighted, MRI-based mouse brain atlas (Ma et al. 2005) containing outlined regions of interests (ROIs). The PET images were subsequently aligned with the CT image containing the brain atlas ROIs. Time-activity curves (TACs), based on concentrations normalised to injected dose per body weight (SUV) were extracted for cortex, cerebellum, hippocampus, midbrain and thalamus.

### Stability test

To test for the stability of the radiolabelled tetrazines, samples were mixed with PBS or plasma and incubated at 37 °C. Samples were taken at 5, 10, 15, 20, 30, 60, 90, 120, 150 and 180 min and analysed with radio-TLC (silica gel 60 F254 (Merck), n-heptane/EtOAc, 40:60). TLC plates were read by a Cyclone Storage Phosphor System (Packard Instruments Co) and quantified with the Optiquant software (version 3.00, Packard Instruments Co) and further analyzed in Graph Pad Prism 9.

## Results

### Radiochemistry

The two labelled compounds, [^18^F]MeTz and [^18^F]HTz, were synthesized from the previously reported[^18^F]F-Py-TFP active ester **4**, Scheme 1 (Syvänen et al. 2020). In short, [^18^F]F-Py-TFP **4** was conveniently produced from Py-TFP **3** and [^18^F]fluoride on the solid phase of an anion exchange column used for trapping and concentrating the [^18^F]fluoride delivered in target water from the cyclotron. Conventional drying and anhydrous conditions, which are typically required for ^18^F-fluorination and nucleophilic substitution, were not needed. The quaternary ammonium precursor **3** was removed by passage over a cation exchange cartridge, eliminating the need for intermediate HPLC-purification. In the final step [^18^F]MeTz or [^18^F]HTz were synthesised using a straight forward amidation with tetrazine propylamines **5a** or **5b**.

**Fig. 1:**
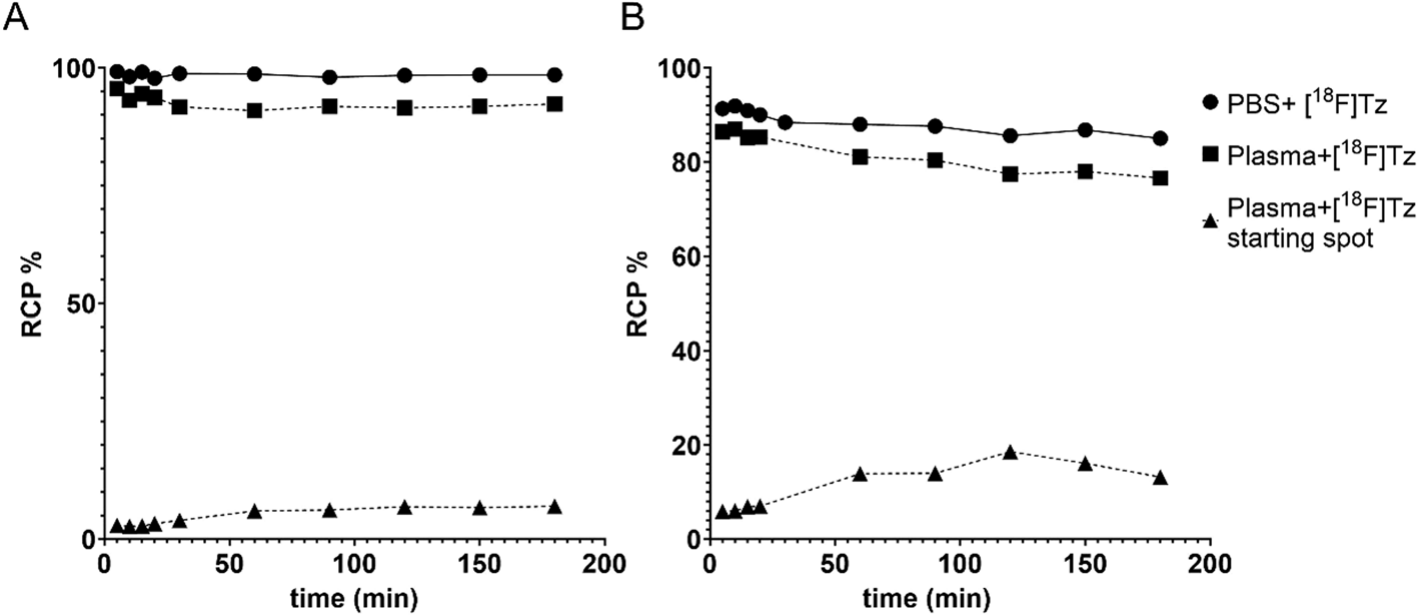
Radiochemical purity of **A** [^18^F]MeTz and **B** [^18^F]HTz measured over 180 min. The stability test in PBS was performed for [^18^F]HTz in the presence of ascorbic acid as stabilizer and for [^18^F]MeTz without ascorbic acid

The methyltetrazine derivative [^18^F]MeTz was obtained with a radioactivity yield of 1.11 ± 0.05 GBq (n = 3), a radiochemical yield of 24% and radiochemical purity of 97% (Coenen et al. 2018). The synthesis of [^18^F]HTz provided similar results, with a radioactivity yield of 1.87 ± 1.04 GBq (n = 3), a radiochemical yield of 22%, and an initial radiochemical purity of 56%, which decreased to 27% within two hours post synthesis. To prevent this rapid degradation of [^18^F]HTz, the compound was formulated with 1.5 mM ascorbic acid in PBS. As a result, the radiochemical purity at end of synthesis increased to 96.5%. The stability in PBS was investigated over 180 min (Fig. 1). [^18^F]MeTz and [^18^F]HTz were 98% and 85% intact after 180 min, respectively. Thus, in comparison, [^18^F]HTz was less chemically stable, despite being formulated with ascorbic acid to reduce decomposition. In vitro plasma stability was also investigated, as an indication of in vivo stability. Both tetrazines were less stable in plasma compared to PBS. After 180 min, 92% of the [^18^F]MeTz was still intact. Radio-TLC-analysis of the [^18^F]MeTz showed that some residual activity remained at the baseline of the TLC plate. After 180 min, 7% of the activity remained at the baseline, possibly indicating binding to plasma proteins. In line with the results in PBS, [^18^F]HTz was also less stable than [^18^F]MeTz in plasma. After 5 min, 86% of [^18^F]HTz was intact, while this number was reduced to 77% at 180 min. Again, some activity remained at the baseline of the TLC plate (13%).

### PET imaging

**Fig. 2:**
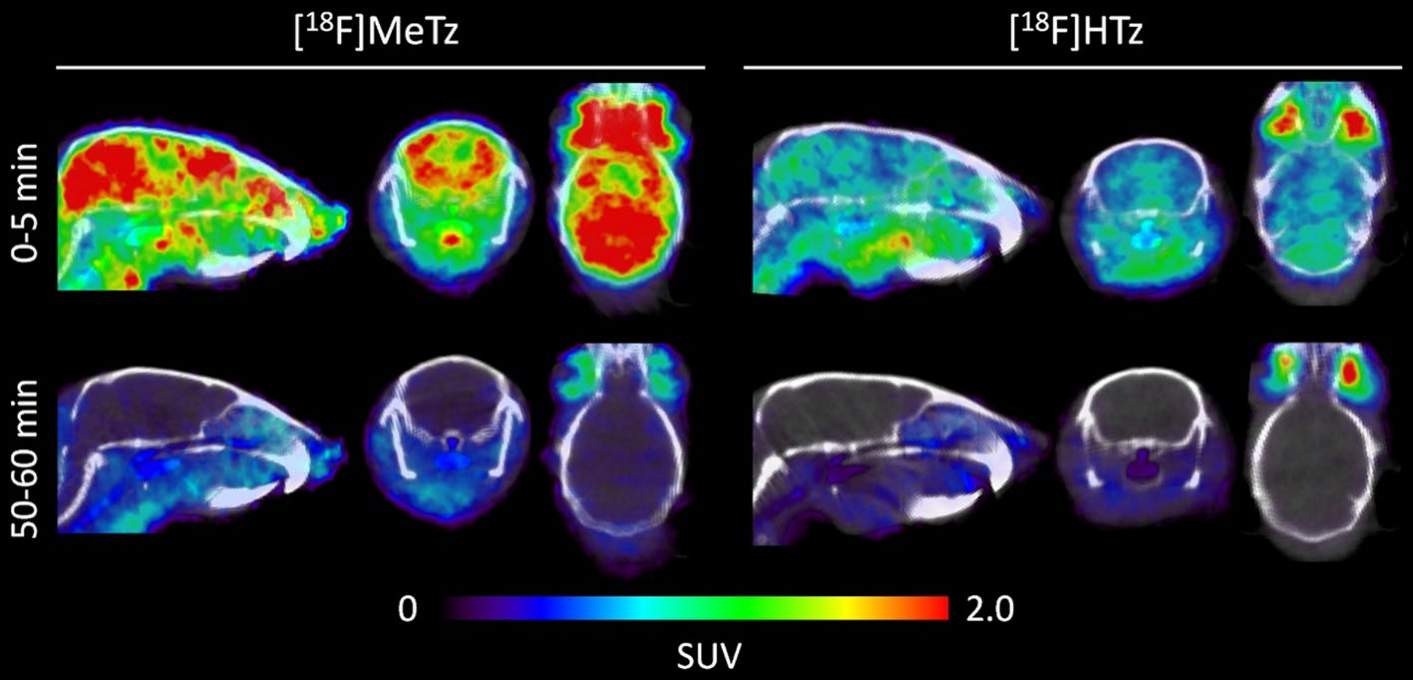
PET images of [^18^F]MeTz and [^18^F]HTz obtained in tg-ArcSwe animals. The images represent the first five min and the last 10 min of the respective scan

PET images of the first 5 min of the scan showed that [^18^F]MeTz entered the mouse brain at higher concentrations than what was observed for [^18^F]HTz (Fig. 2). At the end of the scan, the brains were largely devoid of signal, indicating low remaining concentrations of both tetrazines.

Time-activity curves (TACs) extracted for [^18^F]MeTz and [^18^F]HTz in the whole brain, cortex, hippocampus, and cerebellum indicated a maximum concentration (Cmax) in the whole brain within the first minute, followed by a gradual decline over the 60 min scanning time.

**Fig. 3:**
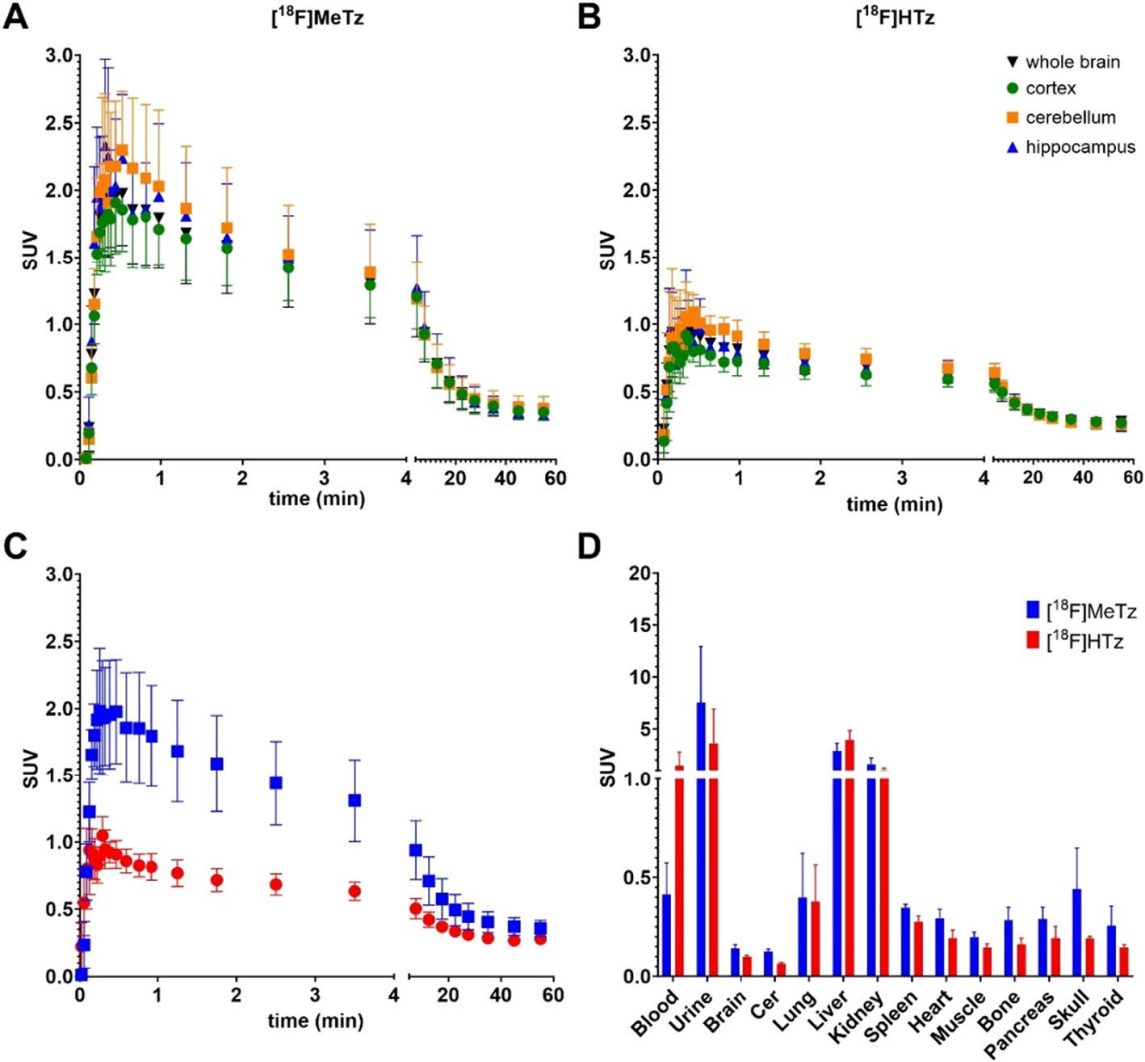
Time-activity curves of selected brain regions of [^18^F]MeTz and [^18^F]HTz. **A** [^18^F]MeTz 0.43 ± 0.12 MBq/g mouse and **B** [^18^F]HTz 0.38 ± 0.06 MBq/gmouse. **C** Comparison of whole brain [^18^F]MeTz and [^18^F]HTz TACs. **D** Biodistribution post-mortem

Similar patterns of uptake were observed in selected brain regions, with slightly higher uptake in the hippocampus and cerebellum compared with the cortex and whole brain (Fig. 3A and B). There was no difference between tg-ArcSwe and WT animals, and thus, for subsequent analysis the animals were pooled to yield n = 4 for each tetrazine. As shown by the PET images, [^18^F]MeTz had a higher brain uptake with a whole brain SUV of 2.0 ± 0.4 at C_max_, compared to 1.0 ± 0.1 for [^18^F]HTz, suggesting that [^18^F]MeTz crossed the BBB and entered the brain more efficiently (Fig. 3C). [^18^F]MeTz also displayed a broader peak than [^18^F]HTz over the first 5 min of the scan, suggesting a somewhat prolonged retention in the brain. At the end of the scan, the whole brain SUVs were 0.36 ± 0.06 for [^18^F]MeTz and 0.28 ± 0.03 for [^18^F]HTz, but this difference was not statistically significant. Thus, even though [^18^F]MeTz entered the brain to a larger extent within the first 5 min, both tetrazines were cleared to a similar concentration at the end of the scan.

The biodistribution was assessed after the animals were euthanized, at 100 min after injection (Fig. 3D). Renal excretion appeared to be the main excretion path for both tetrazines, concluded from the high *post mortem* SUV in urine and kidney. It was also observed that both tetrazines had a noticeable uptake in the liver, suggesting additional metabolism via the liver or binding to liver proteins. Nevertheless, no significant defluorination of either of the tetrazines was expected to have occurred, as the radioactivity in bone was minor as observed in both the *post mortem* biodistribution and in the PET images (Even-Sapir et al. 2007).

## Discussion

The synthesis of [^18^F]MeTz and [^18^F]HTz was straightforward and expands the scope of [^18^F]F-Py-TFP as a labelling precursor, which simplifies the labelling process compared to conventional ^18^F-labelling by avoiding the need for anhydrous reaction conditions and drying of the [^18^F]fluoride (Olberg et al. 2010). The labelled active ester combines a high reactivity towards nucleophilic aromatic substitution with [^18^F]fluoride and the reactive ester functionality for fast coupling with amines. Through the successful reaction with tetrazine propylamine derivatives **5a** and **5b**, the scope of [^18^F]F-Py-TFP as a labelling precursor for ^18^F-labelled tetrazines has also been expanded, complementing the previously reported 6-[^18^F]fluoro-N-(4-(1,2,4,5-tetrazine-3-yl)-benzyl)nicotinamide and the corresponding methyltetrazine analogue (Syvänen et al. 2020; Wegrzyniak et al. 2023; Cheung et al. 2023; Roshanbin et al. 2022). The radiochemical yields and purities achieved for [^18^F]MeTz and [^18^F]HTz were similar and sufficiently high for biological evaluation, while the difference in chemical stability between the two compounds required different formulation strategies. The rapid degradation of [^18^F]HTz post-synthesis was avoided by the addition of ascorbic acid to the formulated product as a stabilizing additive. The differential stability of [^18^F]MeTz and [^18^F]HTz in plasma versus PBS may indicate that metabolism could affect their behaviour in vivo differently. The biodistribution findings, particularly the significant renal excretion and liver uptake, indicate that the compounds are efficiently cleared from blood and other organs, an important aspect of a pretargeting agent aimed for brain imaging.

The PET imaging showed a differential brain uptake between [^18^F]MeTz and [^18^F]HTz, with [^18^F]MeTz demonstrating better BBB penetration possibly due to increased lipophilicity by the added methyl-group (Stéen et al. 2021). The remaining PET signal in the brain at the end of the scan indicate low non-specific binding to brain tissue. Wager et al. (2010, 2016) has described that a molecule targeting the brain needs to show a balanced lipophilicity. It should be lipophilic enough to cross the BBB transcellularly (Chowdhury et al. 2021), but hydrophilic enough to keep the non-specific binding low enough for imaging contrast (Keinänen et al. 2016). This latter point is important, as the brain contains a high percentage of fatty tissue (Chang et al. 2009). For pre-targeting, a good imaging contrast therefore relies on the combination of initial high brain uptake, followed by efficient washout of the tetrazine. Therefore, the relationship between maximum and minimum brain concentrations of the ligand over the 60 min scan time can serve as an important indicator. The C_max_/C_min_ ratio was 5.6 for [^18^F]MeTz and 3.9 for [^18^F]HTz, suggesting that [^18^F]MeTz performed better in this respect. However, in terms of rection kinetics, H-tetrazines generally offer an advantage by exhibiting faster reaction rates in IEDDA reactions compared to methyl-tetrazines, which suggests that [^18^F]HTz could be more beneficial for pre-targeting applications. This potential benefit, however, has yet to be confirmed in vivo by predosing of a compound functionalized with a TCO-group for pre-targeting, which was not performed in the current study.

## Conclusion

In pursuit of developing compounds suitable for pre-targeting applications, two new fluorine-18 labelled tetrazines, [^18^F]MeTz and [^18^F]HTz, were synthesized. This involved labelling of the [^18^F]F-Py-TFP active ester on solid support followed by amidation to incorporate the tetrazine moiety. When studied with PET in mice, both [^18^F]MeTz and [^18^F]HTz were able to cross the BBB and reach the brain, but [^18^F]MeTz entered more efficiently and achieved a higher maximum SUV-value. Both [^18^F]MeTz and [^18^F]HTz were cleared from the blood, and showed low non-specific binding in all brain regions at the end of the experiment. Ex vivo biodistribution studies showed uptake in the liver and kidney due to clearance from the blood. No defluorination was observed in vivo, but [^18^F]MeTz was more stable than [^18^F]HTz in PBS and plasma. Overall, [^18^F]MeTz was more stable and showed the highest brain uptake in combination with efficient clearance from the brain, making it a promising candidate for use in pre-targeted PET imaging.

### Supplementary Information

**Additional file 1:** Supplementary Information.

## Data Availability

The authors declare that the data supporting the findings of this study are available within the paper and its Supplementary Information files. Should any raw data files be needed in another format they are available from the corresponding author upon reasonable request.

## Abbreviations

[^18^F]MeTz: ^18^F-labelled methyl tetrazine
[^18^F]HTz: ^18^F-labelled H-tetrazine
Aβ: Amyloid-beta
BBB: Blood–brain barrier
cLogP: Octanol–water partition coefficient
Cmax: Maximum concentration
CT: Computed tomography
HPLC: High-performance liquid chromatography
IEDDA: Inverse electron demand diels–alder
MRI: Magnetic resonance imaging
NMR: Nuclear magnetic resonance
PET: Positron emission tomography
PBS: Phosphate-buffered Saline
ROI: Region of interest
SUV: Standardized uptake value
TAC: Time-activity curve
TCO: Trans-cyclooctene
TfR: Transferrin receptor
TLC: Thin-layer chromatography
UV: Ultraviolet
WT: Wild-Type
