# Supplementary material for: Synthesis and evaluation of fluorine-18 labelled tetrazines as pre-targeting imaging agents for PET
Source: EJNMMI Radiopharm Chem. 2024 Mar 6;9:21. doi: 10.1186/s41181-024-00250-6 (PMC10917718; doi:10.1186/s41181-024-00250-6)
Supplement: Supplementary file 1 — Additional file 1: Supplementary Information. [file 41181_2024_250_MOESM1_ESM.docx]

Supplementary Information

**Synthesis and evaluation of fluorine-18 labelled tetrazines as pre-targeting imaging agents for PET**

Eva Schlein^1^, Johanna Rokka^1^, Luke R. Odell^2^, Sara Lopes van den Broek^1^, Matthias M. Herth^3, 4^, Umberto M. Battisti^3^, Stina Syvänen^1^, Dag Sehlin^1^, Jonas Eriksson^2, 5*^

*^1^Department of Public Health and Caring Sciences, Uppsala University, 751 85 Uppsala, Sweden*

*^2^Department of Medicinal Chemistry, Uppsala University, 751 23 Uppsala, Sweden*

*^3^Department of Drug Design and Pharmacology, University of Copenhagen, 2100, Copenhagen, Denmark*

*^4^Department of Clinical Physiology, Nuclear Medicine & PET, Rigshospitalet Copenhagen University Hospital, Blegdamsvej 9, 2100 Copenhagen, Denmark*

*^5^PET Centre, Uppsala University Hospital, 751 85 Uppsala, Sweden*

**Corresponding author:**

Jonas Eriksson

E-mail: [jonas.eriksson@ilk.uu.se](mailto:yazkim@ncgg.go.jp)

**Synthesis of Py-TFP 3**

**Scheme 1**. a) 2,3,5,6-tetra-fluorophenol, N’,N’-dicyclohexylcarbodiimide, acetonitrile, RT, 24 h.
b) trimethylamine, tetrahydrofuran, RT, 5 h. c) trimethylsilyl triflate, dichloromethane

Py-TFP **3** was synthesized as previously described ^29^ with minor modifications. Commercially available 6-chloronicotinic acid **1** (2.01 g, 12.7 mmol), 2,3,5,6-tetra-fluorophenol (2.21 g, 19 mmol), and N’,N’-dicyclohexylcarbodiimide (2.62 g, 12.6 mmol) were dissolved in acetonitrile (100 mL) and the mixture was stirred at room temperature for approximately 24 hours. The resulting solid product was isolated by filtration, then dried under high vacuum to remove residual solvents and volatiles. The residue was dissolved in a small amount of hot hexane and immediately filtered. The filtrate was allowed to stand at 4 °C overnight filtered and washed with cold hexane. The resulting crude 6-chloronicotinic acid 2,3,5,6-tetrafluorophenyl ester **2** (1.58 g, 5.2 mmol) was used directly in the next step without further purification.

Activated ester **2** (1.5 g, 4.9 mmol) was dissolved in 15 mL of 1 M trimethylamine in THF and the mixture was stirred at room temperature for 5 hours. The resulting suspension was centrifuged, washed with cold ethyl acetate and cold dichloromethane, and dried under reduced pressure. The solid material was then suspended in dichloromethane (100 mL) and stirred under nitrogen atmosphere at room temperature while trimethylsilyltriflate (2.4 mL) was slowly added. After the addition, the mixture was stirred for 20 min and then filtered to recover the solids. After the volatiles were removed from the solids under reduced pressure, the material was washed with diethyl ether. Subsequently, the solvent was removed under reduced pressure, yielding *N,N*-trimethyl-5-((2,3,5,6-tetrafluorophenoxy)-carbonyl)pyridin-2-aminium trifluoromethanesulfonate Py-TFP **3** (1.57 g, 3.28 mmol) as a white powder.

**^1^H NMR data for 2**

**^1^H NMR data for 8**

**^1^H and ^13^C NMR data for 10a and 10b**

FMeTz **10a,** 6-fluoro-N-(3-(4-(6-methyl-1,2,4,5-tetrazin-3-yl)phenoxy)propyl)nicotinamide

^1^H NMR (400 MHz, DMSO-*d*_6_) δ 8.79 (t, *J* = 6.2 Hz, 1H), 8.70 (d, *J* = 2.5 Hz, 1H), 8.42 (d, *J* = 8.9 Hz, 2H), 8.38 (dd, *J* = 8.1, 2.5 Hz, 1H), 7.30 (dd, *J* = 8.6, 2.7 Hz, 1H), 7.21 (d, *J* = 8.9 Hz, 2H 2H), 4.19 (t, *J* = 6.5 Hz, 2H), 3.48 (q, *J* = 6.2 Hz, 2H), 2.96 (s, 3H), 2.05 (quint, *J* = 6.5 Hz, 2H).

^13^C NMR (101 MHz, DMSO-*d*_6_) δ 166.5, 165.1 (d, *J* = 284 Hz), 163.0, 162.1, 147.3, 141.4, 129.2, 128.7, 124.9, 115.4, 109.3 (d, *J* = 38 Hz), 65.7, 36.3, 28.6, 20.7.

FHTz **10b**, N-(3-(4-(1,2,4,5-tetrazin-3-yl)phenoxy)propyl)-6-fluoronicotinamide

^1^H NMR (400 MHz, DMSO-*d*_6_) δ 10.49 (s, 1H), 8.80 (t, *J* = 5.6 Hz, 1H), 8.70 (ddd, *J* = 2.6, 1.7, 0.8 Hz, 1H), 8.49 – 8.43 (m, 2H), 8.38 (ddd, *J* = 8.6, 7.8, 2.5 Hz, 1H), 7.30 (ddd, *J* = 8.6, 2.7, 0.7 Hz, 1H), 7.26 – 7.19 (m, 2H), 4.20 (t, *J* = 6.2 Hz, 2H), 3.51 – 3.45 (m, 2H), 2.06 (quintet, *J* = 6.2 Hz, 2H).

^13^C NMR (101 MHz, DMSO--*d*_6_) δ 165.2, 163.4, 162.5, 157.7, 147.3 (d, *J* = 16.3 Hz), 141.5 (d, *J* = 9.1 Hz), 129.7, 128.8, 124.0, 115.5, 109.4, 65.8, 36.3, 28.7.
